# Bacteriological Profile of Diabetic Foot Ulcer With Special Reference to Biofilm Formation

**DOI:** 10.7759/cureus.80974

**Published:** 2025-03-21

**Authors:** Nitya Tirumala, Leela Rani K

**Affiliations:** 1 Medicine, Vydehi Institute of Medical Sciences and Research Centre, Bangalore, IND; 2 Microbiology, Vydehi Institute of Medical Sciences and Research Centre, Bangalore, IND

**Keywords:** antibiotic resistance (abr), bacteriological profile, biofilm formation, diabetic foot ulcer (dfu), multidrug-resistant organisms

## Abstract

Introduction

Diabetes mellitus is a metabolic disorder characterized by abnormally high sugar levels in the blood for prolonged periods of time. The world’s largest number of diabetics resides in India, making it the world’s diabetic capital, with a diabetic foot ulcer (DFU) incidence of around 8-17%. Due to the polymicrobial and multidrug resistant (MDR) nature of DFUs, antimicrobial susceptibility testing is of high importance to help treat patients effectively and prevent the development of MDR bacteria. The ability to form biofilms is a significant additive to virulence of an organism that causes an able strain to be resistant to more antibiotics as compared to a free-living strain, thereby further delaying the healing of DFUs.

Materials and methods

This study included 74 samples collected from patients with DFUs, out of which 69 (93.24%) yielded growth on culture. Gram staining was done for the direct microscopy, isolation, and determination of organism, and the detection of biofilm formers using Congo Red Agar plates. Data were tabulated and statistically analyzed.

Results

Out of 74 samples collected, 69 (93.24%) yielded growth on culturing, with 5 (6.76%) cultures coming back negative. On Gram staining, 42 (56.8%) samples showed Gram-positive cocci and 53 (71.6%) showed Gram-negative bacilli. On isolating organisms from the samples, 16 (21.7%) samples had *Pseudomonas aeruginosa*, followed by *Klebsiella pneumoniae* at 13 (17.6%) and *Proteus mirabilis* and *Escherichia coli* at 11 each (14.9%), indicating a Gram-negative bacteria predominance. Of the Gram-positive bacteria isolated, *Staphylococcus aureus* stands at two (2.7%) and *Streptococcus pyogenes* at one (1.4%). The prevailing monomicrobiality could be attributed to antibiotic administration prior to sample collection. On antibiotic sensitivity of organisms tested against each antibiotic, more than 75% of tested samples were resistant to ampicillin, cefuroxime, and erythromycin, and around 70% and more of tested samples were sensitive to ertapenem, meropenem, amikacin, gentamicin, benzylpenicillin, vancomycin, and clindamycin.

Out of the 69 positive cultures, 29 (42.03%) yielded biofilm formers. *Pseudomonas aeruginosa* was the predominant biofilm former with 10 (34.48%) of 29 of total biofilm-forming isolates, followed by *E. coli* and *K. pneumoniae* with five each (17.24%) and *Proteus mirabilis* with three samples (10.34%). Regarding strains forming biofilms according to bacterium species, *S. aureus* was predominant, with 100% (two out of two samples) of isolates forming biofilms. *Staphylococcus aureus* was followed by *Pseudomonas aeruginosa* with 62.5% (10 out of 16 samples), *Acinetobacter baumannii* (two out of four samples) and *Enterobacter cloacae* (one out of two samples) with 50% each, *E. coli* with 45.45% (5 out of 11 samples), and *K. pneumoniae* with 38.46% (5 out of 13 samples) of isolates forming biofilms. It should be noted that only two *S. aureus*, four *A. baumannii,* and two *Enterobacter cloacae* samples were isolated in comparison to a larger number of Gram-negative bacteria. This study has found that 100% of multidrug-resistant organisms (MDROs) are biofilm formers.

Conclusion

The ability to form biofilms adds immensely to the virulence and antibiotic resistance. Detection of biofilm formers is non-invasive and convenient to measure and would help provide insight into antibiotics to be administered to the patient, thereby reducing development of MDROs and reducing healing time.

## Introduction

Diabetes mellitus is a metabolic disorder characterized by abnormally high sugar levels in the blood for prolonged periods of time. Type 1 diabetes mellitus arises due to autoimmunity targeting the insulin-producing beta cells, leading to insulin deficiency. Type 2 diabetes mellitus is a heterogenous set of disorders marked by varying levels of insulin resistance, impaired insulin secretion, and enhanced hepatic glucose production. It is caused either due to insufficient production of insulin by the pancreas or due to impaired response of the body to the produced insulin [1]. Other characteristics include frequent urination, increased thirst, and hunger.

The world’s largest number of diabetics resides in India, making it the world’s diabetic capital. The number of diabetics is expected to increase from 171 million in 2000 to 366 million in 2030 [2]. One of the most common complications of diabetes mellitus, among others such as cardiovascular diseases, strokes, and retinopathy, is foot ulcers leading to non-traumatic amputation of the limbs. The primary, non-traumatic causes for amputation include diabetic foot ulcer (DFU) and diabetic foot infections (DFIs). Each year, around 18.6 million individuals globally are affected by DFU, with 1.6 million of those cases occurring in the United States [3]. Around 12%-25% of diabetics across the world are at the risk of developing foot ulcers [4-6], with the major underlying causes of the DFUs being peripheral neuropathy and micro- and macro-angiopathy resulting in ischemia. The cause of DFUs is multifactorial, making it complex. In patients with diabetic polyneuropathy and/or angiopathy, a combination of increased pressure on the foot and systemic factors such as impaired wound healing contributes to chronic foot lesions. Epidemiological data show that neuropathy alone accounts for approximately 50% of diabetic foot syndrome cases. Peripheral arterial occlusive disease is responsible for 15%, while 35% of foot ulcers result from a combination of both neuropathy and angiopathy [7].

In India, the incidence of DFUs ranges from 8% to 17% [8]. Management and treatment of diabetic ulcers is very important as the high levels of blood sugar levels and lowered immunity impair the healing of these ulcers. Factors aggravating the already compromised healing prevalent in India could be walking barefoot, inadequate facilities for diabetic care, low socioeconomic status, and illiteracy [9,10].

DFIs are predominantly polymicrobial rather than monomicrobial and are multidrug-resistant (MDR) [8]. To be able to increase the success rate of healing of DFUs, sound knowledge of the bacteriological profile of the ulcers is required. Some of the commonly found aerobes are Gram-positive *Staphylococcus aureus*, *Enterococcus*, and Gram-negative *Pseudomonas aeruginosa*, *Escherichia* *coli*, *Klebsiella *spp., *Proteus* spp., [8,11] and anaerobes are *Peptostreptococcus *spp. and *Bacteroides* spp. [12]. Testing of antibiotic sensitivity of these bacteria is important so as to start the patient on appropriate antibiotic at the earliest. It also prevents the development of antibiotic-resistant bacteria.

Certain bacteria tend to be biofilm formers. Accumulation of either mono- and/or poly-microbial microorganisms to form sessile, sedentary communities is known as a biofilm [9]. They are formed when these microorganisms irreversibly attach themselves to the surface and begin to secrete extracellular polymers that facilitate matrix formation. These result in phenotypical changes in the strains of bacteria with respect to growth rate and gene transcription. Biofilm-associated strains are more resistant to antibiotics than free living bacteria as their exposure to the antibiotic is so slow that they tend to develop resistance to it [13].

The delayed healing in DFUs is attributed primarily to host factors, which include reduced immunity and impaired healing. The tendency of the bacteria to form a biofilm, leading to delayed healing, has been studied. The current practice involves culture and sensitivity of a sample from the foot ulcer and administration of an appropriate antibiotic. However, the tendency of the bacteria to form a biofilm is not routinely evaluated. Hence, this study aims to look at biofilm formation among the pathogens isolated from all the cases of DFU.

## Materials and methods

The study is a cross-sectional study conducted on 74 cases of DFUs over a period of two months in the Department of Microbiology in association with the Department of Surgery of a tertiary care center. The study included patients with diabetes mellitus, presenting with diabetic foot ulcers of more than three months' duration. All patients were over the age of 18 years, and only consenting patients were included in the study. Patients with comorbidities such as HIV infection and chronic venous insufficiency, pregnant patients, patients less than 18 years of age, and patients who did not consent to the study were excluded from the study.

Samples were collected after obtaining consent of the patients and under aseptic conditions. Detailed history taking was followed by clinical examination. Samples were obtained on the day of admission and/or during dressing changes. Sterile gloves were worn, and using a sterile swab from a swab container, samples were taken from the ulcer's base after cleaning it with normal saline. Pus and soft tissue samples were collected following proper cleaning. The samples were promptly transported to the microbiology laboratory.

In the Department of Microbiology, samples are submitted for culture sensitivity testing and biofilm formation analysis. Direct microscopy of the swabs received was done using Gram stain. Then swabs received were cultured on blood agar and McConkey agar, and the plates were incubated at 37°C to isolate aerobic bacteria present in the DFU. The colonies that grew on the culture plates were identified by established biochemical tests and Clinical and Laboratory Standards Institute (CLSI) guidelines [14]. Sensitivity of the pathogen to the antibiotics was documented using the disc diffusion method.

The biofilm formation was detected using the Congo Red method. Culture plates were prepared using brain heart infusion agar (35 g/L), Congo red stain (0.8%), sucrose (5g/L), and Agar powder. Plates were then dried and streaked and left to intubate at 37°C. Congo red forms bonds with the polysaccharides present in the biofilm matrix, creating colored complexes. On Congo red agar plates, colonies that appear black and dry indicate strong biofilm production, while red colonies suggest the absence of biofilm production. This provides a simple and economical way of screening for biofilm-forming organisms.

The results obtained were statistically analyzed.

## Results

A total of 74 samples from 74 patients with DFUs were included in this study. Majority of patients were between 60 and 70 years of age. Median age distribution in this study was 63 years (Table 1). Of the patients, 51 were male and 23 were female. Significant number of samples showed abundant pus cells in the background, with majority of them being Gram-positive cocci or Gram-negative bacilli. However, some of them showed mixed flora. Of the 74 samples submitted, 18 (24.32%) showed the presence of Gram-positive cocci on Gram stain, and 61 (82.43%) showed the presence of Gram-negative bacilli on Gram stain.

**Table 1:** Median age distribution

	Median	25th Percentile	75th Percentile
Age	63	52	68

A total of 74 samples were processed, which yielded 69 (93.24%) positive cultures. Five (6.76%) samples came back culture-negative. All the culture-positive samples were monomicrobial. *Pseudomonas aeruginosa *was the predominant organism, followed by *Klebsiella pneumoniae*. Table 2 shows the frequency and percentage of the organisms isolated from the samples.

**Table 2:** Organisms isolated in the cultures

Organism Isolated	Frequency	Percentage
Pseudomonas aeruginosa	16	21.7
Klebsiella pneumoniae	13	17.6
Escherichia coli	11	14.3
Proteus mirabilis	11	14.3
Nil	5	6.8
Acinetobacter baumannii	4	5.4
Morganella morganii	3	4.1
Staphylococcus aureus	2	2.7
Serratia marcescens	2	2.7
Enterobacter cloacae	2	2.7
Proteus penneri	1	1.4
Serratia fonticola	1	1.4
Streptococcus pyogenes	1	1.4
Providencia rettgeri	1	1.4
Enterococcus faecalis	1	1.4
Total	74	100

Table 3 shows the frequency and percentage of the isolated organisms that are MDR.

**Table 3:** Frequency and percentage of MDROs *Methicillin-resistant *Staphylococcus aureus* MDRO, multidrug-resistant organism

Organism Isolated	Total Number Isolated	Total MDRO	Percent MDRO
Pseudomonas aeruginosa	16	3	18.75
Klebsiella pneumoniae	13	4	30.76
Escherichia coli	11	0	0
Proteus mirabilis	11	0	0
Acinetobacter baumannii	4	2	50
Morganella morganii	3	1	33.33
Staphylococcus aureus	2	1*	50
Serratia marcescens	2	0	0
Enterobacter cloacae	2	1	50
Proteus penneri	1	0	0
Serratia fonticola	1	0	0
Streptococcus pyogenes	1	0	0
Providencia rettgeri	1	0	0
Enterococcus faecalis	1	0	0
Total	69	12	

Biofilm formation was seen in 29 of the 69 culture-positive samples, constituting 42.08%. Biofilms were formed predominantly by Pseudomonas aeruginosa (34.48%, 10 samples). Table 4 shows the frequency and percentage of the organisms that form biofilms.

**Table 4:** Organisms forming biofilm

Organism Isolated	Total Number Isolated	Number of Isolates Forming Biofilm	Percentage of Isolates Forming Biofilm	Percentage of Each Organism Forming Biofilms Among the Total Biofilm Formers
Pseudomonas aeruginosa	16	10	34.48	62.5
Klebsiella pneumoniae	13	5	17.24	38.46
Escherichia coli	11	5	17.24	45.45
Proteus mirabilis	11	3	10.34	27.27
Acinetobacter baumannii	4	2	6.89	50
Staphylococcus aureus	2	2	6.89	100
Enterobacter cloacae	2	1	3.44	50
Morganella morganii	3	1	3.44	33.33
Serratia marcescens	2	0	0	0
Serratia fonticola	1	0	0	0
Proteus penneri	1	0	0	0
Providencia rettgeri	1	0	0	0
Enterococcus faecalis	1	0	0	0
Streptococcus pyogenes	1	0	0	0
Total	69	29	100	

Table 5 shows the frequency of isolated multidrug-resistant organisms (MDROs) forming biofilm.

**Table 5:** MDROs forming biofilm *Methicillin-resistant *Staphylococcus aureus* MDRO, multidrug-resistant organism

Organism Isolated	Number of MDROs	Number of MDROs Forming Biofilm
*Klebsiella pneumoniae*	4	4
*Pseudomonas aeruginosa*	3	3
*Acinetobacter baumannii*	2	2
*Staphylococcus aureus*	1*	1
*Enterobacter cloacae*	1	1
*Morganella morganii*	1	1
Total	12	12

Around 50% of the isolated organisms tested against the antibiotics were resistant to ticarcillin (11 out of 19 samples, 57.9%), amoxycillin clavulanate (21 out of 45 samples, 46.7%), ceftazidime (10 out of 19 samples, 52.6%), tigecycline (29 out of 59 samples, 49.2%), colistin (30 out of 61 samples, 49.2%), and trimethoprim (27 out of 48 samples, 56.3%). More than 60% of the isolated bacteria were resistant to ampicillin (36 out of 41 samples, 87.8%), cefuroxime (36 out of 46 samples, 78.3%), ceftriaxone (28 out of 46 samples, 60.9%), ciprofloxacin (39 out of 65 samples, 60%), levofloxacin (15 out of 24 samples, 62.5%) and erythromycin (four out of four samples, 100%). Around 87.80% and 100% of tested isolated strains were resistant to ampicillin (36 out of 41 samples) and erythromycin (four out of four samples), respectively.

Around 60% were sensitive to piperacillin (36 of 61 samples), cefperazone (42 of 62 samples), cefepime (37 of 64 samples), ertapenem (44 of 63 samples), and meropenem (44 of 63 samples). Also, 75% of strains isolated and tested against benzylpenicillin (three of four samples) and clindamycin (three of four samples) and 77% tested against gentamicin (45 of 58 samples) and amikacin (45 of 58 samples) showed sensitivity to the same. Overall, 100% showed sensitivity to vancomycin (four of four samples) and linezolid (four of four samples).

## Discussion

Diabetes mellitus along with its long-term complications are a major concern. Diabetes-related foot complications are the single most important cause of morbidity and hospitalization among these patients. The associated complicating factor of peripheral vascular disease caused by the disease itself makes majority of DFU cases asymptomatic until later in the course of disease evolution [15]. Bacteria that grow and thrive in the biofilms are sheltered and protected from host immune responses and antimicrobial agents. The biofilm formation by the bacteria has been strongly associated with marked antibiotic resistance and chronic recurrent infections [16].

Diabetes mellitus is a common disease in India, whose numbers seem to be increasing alarmingly. The prevalence rate of this disease is around 12-17% in the urban population and 2.5% in the rural population [16]. Non-traumatic lower limb amputation mostly due to DFUs and DFIs is one of the most dreaded complications of this condition [16].

The incidence of foot ulcers in diabetic patients ranges from 8% to 17% in India [17]. Peripheral neuropathy, another associated complication of diabetes mellitus, seems to contribute significantly to the development of ulceration. Factors aggravating the already compromised healing could be walking barefoot, inadequate facilities for diabetic care, low socioeconomic status, and illiteracy [5,6]. Diabetic patients with pre-ulceration, callosities, and deformity are at an increased risk [17].

The annual incidence of DFUs is projected to be 1.0-4.1%, while the lifetime risk is around 25% [18]. A common complication is infection, which results in the need for distal limb amputation if left untreated [5].

Diabetic neuropathy and micro- and or macrovascular disease are the two main risk factors that cause DFU [19]. Impaired circulation appears to limit the access of phagocytic cells to infected area. This also results in poor concentration of antibiotics in infected tissue [9]. Hence, diabetic foot wounds are usually prone for infections.

In a study by conducted by Banu et al., all samples yielded monomicrobial isolates. Of the isolates, 24.4% (20 organisms) were found to be Gram-positive while 75.6% (62 organisms) were Gram-negative. *Staphylococcus aureus* and *Escherichia coli* were the commonly isolated organisms (24.40%, 20 organisms each), followed by *Pseudomonas aeruginosa* (17.07%, 14 organisms). Biofilm formation was detected in 46.3% of the isolates (38 isolates). *Staphylococcus aureus *was the predominant biofilm former, with 38.8% (15 out of 20 isolates) of the isolates testing positive for biofilm formation. *Staphylococcus aureus *was followed by *Pseudomonas aeruginosa* with 26% (10 out of 14 isolated) [8].

In a study by Jain and Barman, bacterial isolates were obtained from 150 persons with DFUs. The age group of these persons ranged from 35 to 80 years and, the maximum number of persons with DFUs were in the age group of 60-65 years. Among the isolates, Gram-negative bacilli were isolated in 59% (106 organisms) and Gram-positive cocci in 41% (73 organisms) of cases. The most common isolate was *Staphylococcus *spp in 25% (46 isolated), followed by *Escherichia coli *in 20% (37 isolated) of the cases [9].

In a study conducted by Bansal et al., samples collected post-antibiotic therapy were found to be polymicrobial in nature in 16 (26.22%) out of 61 patients. In this study, *Pseudomonas aeruginosa *among the Gram-negative (21.67%, 31 samples) and *Staphylococcus aureus* among the Gram-positive (18.88%, 27 samples) were the predominantly isolated organisms [10].

In a study by Otta et al., 240 aerobic bacteria were isolated from the DFUs. *Staphylococcus aureus* were the most common aerobic bacteria isolated. Also, 77.8% were methicillin-resistant (56 isolates), while 42.1% (9 isolates) of the Gram-negative *Enterobacteriaceae *were extended-spectrum beta-lactamase (ESBL) positive. *Klebsiella* spp. was the highest ESBL producer, whereas *Acinetobacter* spp. was the highest metallo-beta-lactamase producer. Linezolid, teicoplanin, and vancomycin were the most sensitive drugs for *Staphylococcus *spp. Gram-negative isolates were mostly sensitive to cefoperazone-sulbactam and imipenem. *Pseudomonas* spp. was mostly sensitive to imipenem and piperacillin-tazobactam, whereas *Acinetobacter* spp. was sensitive to netilmicin and levofloxacin [4].

A total of 74 samples from 74 patients of DFUs were assessed in this study. Maximum number of patients were between 60 and 70 years of age. All the samples were found to be monomicrobial in this study in contrast to the polymicrobial samples in some of the other studies [20]. Most of the patients included in this study presented with chronic foot ulcers that were earlier treated with antibiotics. This could be the reason for the monomicrobial yield in the cases in this study.

Overall, 56.8% (42) of the cases showed Gram-positive cocci, and 71.6% (53 cases) of the cases showed Gram-negative bacilli. 6.8% (5 samples) of the cases showed no growth. The findings of Bansal et al. [10] showed that 76% (119 organisms) of the microbes were Gram-negative and 24% (38 organisms) were Gram-positive. Similar findings are documented by the study conducted by Banu et al. [8], who found 24.4% (20 organisms) to be Gram-positive and 75.6% (62 organisms) to be Gram-negative. The predominance of Gram-negative organisms has been noted in several studies.

The commonest pathogens isolated were *Pseudomonas aeruginosa* (21.7%, 16 of 74 samples), followed by *Klebsiella pneumonia* (17.6%, 13 of 74 samples) and *Escherichia coli* along with *Proteus mirabilis* (14.9%, 11 each of 74 samples). These organisms were also seen commonly in the study conducted by Banu et al. [8] and Bansal et al. [10]. The Gram-negative organisms that have been identified commonly in our study were found to show variable sensitivity in the antibiogram: 31% (19 out of 61 samples) of them were found to be resistant to piperacillin, 60.86% (28 out of 46 samples) to ceftriaxone, 39.06% (25 out of 64 samples) to cefepime, and 26.98% (17 out of 63 samples) to meropenem. Gram-positive cocci constituted smaller portion of the sample size and were found to be 100% (four out of four samples) sensitive to linezolid.

Biofilm formation was seen in 29 of the 69 culture-positive samples, constituting 39.18%. *Staphylococcus aureus *was predominant, having 100% (two out of two) of isolates forming biofilms. *Staphylococcus aureus *was followed by *Pseudomonas aeruginosa* with 62.5% (10 out of 16), *Acinetobacter baumannii* and *Enterobacter cloacae *with 50% (two of four and one of two, respectively), *Escherichia coli *with 45.45% (5 out of 11), and *Klebsiella pneumoniae *with 38.46% (5 out of 13) of isolates forming biofilms. It should be noted that only two cases of *Staphylococcus aureus*, four cases of *Acinetobacter baumannii*, and two cases of *Enterobacter cloacae* were isolated in comparison to a larger number of Gram-negative bacteria.

This study found that 100% (12 samples) of MDROs were biofilm formers. The literature review shows a wide variation in the percentage of cases showing biofilm formation. In the study conducted by Banu et al. [8], 46.3% (37 samples) of MDR isolates showed biofilm formation.

This study has attempted to determine the aerobic bacteriological profile of DFU patients and correlate formation of biofilm with the isolated pathogens. However, the limitation of this study is that fungal infections and anaerobic bacteria infections were not identified.

## Conclusions

Both Gram-positive and Gram-negative organisms cause DFU infections. The organisms causing chronic DFUs can be MDR. Such MDR pathogens were also found to be biofilm-forming organisms. Therefore, screening for biofilm formation, along with the antibiotic sensitivity assessment, can be considered to be performed as a routine procedure in chronic diabetic ulcers. This will help in instituting effective treatment strategies for these patients.
